# Kasabach-Merritt Syndrome in Neonates: A Case of Complexity and Care

**DOI:** 10.7759/cureus.88929

**Published:** 2025-07-28

**Authors:** Abdessamad Lalaoui, Khalid Abi el Aala, Zineb Daraoui, Ghizlane Kassal, Fatiha Bennaoui, Maryem Aboudourib, Ouafa Hocar, Sara Ouassil, Ibtissam Zouita, Nadia El Idrissi Slitine, Fadl Mrabih Rabou Maoulainine

**Affiliations:** 1 Neonatal Intensive Care Unit, Mohammed VI University Hospital Center, Marrakech, MAR; 2 Research Center for Childhood, Health and Sustainable Development, Cadi Ayyad University, Marrakech, MAR; 3 Dermatology and Venerology Department, Mohammed VI University Hospital Center, Marrakech, MAR; 4 Radiology Department, Mohammed VI University Hospital Center, Marrakech, MAR

**Keywords:** chemotherapy, corticotherapy, kasabach-merrit syndrome, microangiopathy, neonatal respiratory distress

## Abstract

Kasabach-Merritt syndrome (KMS) is a rare and potentially life-threatening pediatric coagulopathy characterized by thrombocytopenia, microangiopathic hemolytic anemia, and coagulation abnormalities. Clinically, it presents as a reddish-purple mass resembling a hemangioma. Diagnosis relies on a combination of clinical and biological assessments, sometimes supplemented by biopsy to confirm the hemangioma.

We present the case of a one-day-old male neonate with an angioma affecting the right hemothorax and upper limb, accompanied by thrombocytopenia. The patient responded favorably to treatment with corticosteroids, chemotherapy, and aspirin. Therapeutic strategies were carefully evaluated and discussed.

## Introduction

Kasabach-Merritt syndrome (KMS) is a rare and potentially life-threatening coagulopathy that primarily affects infants. Initially described in 1940 by Kasabach and Merritt, the syndrome was first recognized in a newborn presenting with a giant capillary hemangioma and thrombocytopenic purpura [1]. Over time, it has become evident that KMS is characterized by a unique constellation of clinical features, including thrombocytopenia, consumptive coagulopathy, microangiopathic hemolytic anemia, and a distinctive vascular tumor [1].

Unlike benign capillary hemangiomas, which typically regress in childhood and pose primarily cosmetic concerns, KMS-associated vascular tumors exhibit aggressive growth patterns and significant systemic complications. The underlying pathophysiology is driven by platelet and fibrinogen consumption within intralesional thrombosis, leading to coagulation abnormalities and an increased risk of severe bleeding [2]. The lesions, while often superficial and solitary, can extend to internal organs such as the liver, further exacerbating clinical severity. In extreme cases, high-volume arteriovenous shunting may result in cardiac failure, and life-threatening hemorrhages, including intracranial bleeding, contribute to mortality rates as high as 30% [2].

KMS typically manifests in neonates and infants younger than six months, presenting as a large, reddish-purple vascular lesion associated with varying degrees of bleeding tendencies and, in some cases, profound anemia [1]. Diagnosis is confirmed through imaging techniques that assess tumor extent and systemic involvement. Due to the syndrome’s substantial risks-including severe hemorrhage, thrombotic events, tumor-related compression, and potential cardiac complications-early recognition and multidisciplinary management are critical in optimizing patient outcomes [3,4].

This study examines a newly diagnosed case of KMS, providing an in-depth exploration of its clinical presentation, disease progression, and associated complications. In addition, we conduct a comprehensive review of existing literature to highlight the challenges in diagnosing and managing KMS, with a focus on current therapeutic strategies and emerging approaches aimed at improving survival and long-term prognosis.

## Case presentation

A one-day-old male neonate was admitted to the Neonatal Intensive Care Unit (NICU) of the pediatric department at Mohamed VI University Hospital in Marrakesh. He was born to second-degree consanguineous parents. His 41-year-old mother had gestational diabetes, which was managed with a balanced diet during pregnancy. No risk factors for early neonatal infection were identified. The baby was delivered at full term via Caesarean section due to a scarred uterus, with the pregnancy estimated at 38 weeks based on first-trimester ultrasound findings. An antenatal diagnosis had revealed a thoracic mass.

At birth, the neonate exhibited mild respiratory distress, characterized by subcutaneous chest indrawing and a heterogeneous erythematous mass with an orange-peel texture, affecting the right hemithorax and upper limb. Notably, there was no fever or other associated symptoms.

Clinical examination revealed a well-perfused newborn with a pink complexion, spontaneous movements, and stable vital signs: heart rate of 127 beats per minute, respiratory rate of 46 breaths per minute, temperature of 36°C, weight of 3.9 kg, height of 47 cm, and a head circumference of 34 cm.

**Figure 1:**
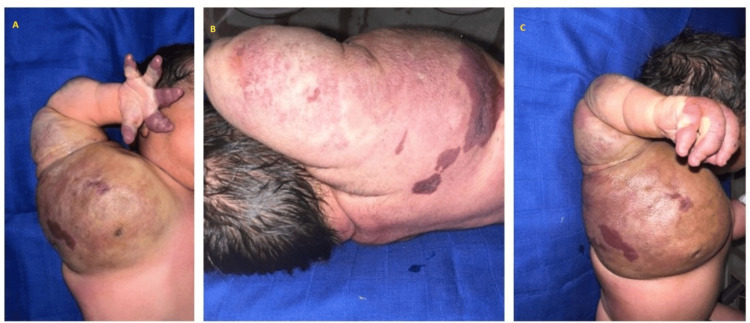
Mass involving the right upper limb and hemithorax

Assessment of the spine and limbs identified a warm, purplish-red, firm, non-fluctuant swelling with heterogeneous characteristics. The mass significantly restricted movement in the shoulder, elbow, wrist, and fingers. No congenital malformations were observed (Figure 1).

A coagulation profile identified thrombocytopenia with a platelet count of 30,000/mm³, occurring without anemia or other hematological abnormalities. Prothrombin time was notably decreased (49%), while D-Dimer and antithrombin III levels remained within normal ranges. Elevated aminotransferase (GOT) activity was observed (243 IU), accompanied by hypercalcemia (130 mg/L) and dyslipidemia, characterized by total cholesterol of 3 mmol/L, triglycerides of 4 mmol/L, and LDL cholesterol of 3.2 mmol/L (refer to Table 1).

**Table 1:** Summary of laboratory Findings. GOT: serum glutamic oxaloacetic transaminase; LDLc: LDL cholesterol

Variables	Day 1	2 months	Normal Range
Leukocytes	10340	11000	7690-13120
Hemoglobin (g/dL)	16	12	12-16
Platelets	30000	255000	140000-238000
TP %	49	90	70-100
GOT (U/l)	243	50	20-67
Anti Thrombin III (U/ml)	0.2	0.19	0.14-0.62
Calcium (mg/dl)	13	9.1	8.5-11
Cholesterol total (mg/dl)	116	100	<170
Triglycerides (mg/dl)	354	120	19-174
LDLc (mg/dl)	120	95	<110

Chest X-ray showed cardiomegaly with a cardiothoracic index of 0.64. Trans-thoracic echocardiography revealed an enlarged left ventricle with 8 mm septal hypertrophy and a 10 mm wall thickness, but normal ejection fraction and no clinical signs of heart failure.

Soft-tissue ultrasound identified a hypervascularized hemangioma extending from the hemithorax to the hand, confirmed by Doppler imaging. A right paracardiac mass with similar characteristics was also noted. Abdominal ultrasound indicated mild hepatosplenomegaly.

**Figure 2:**
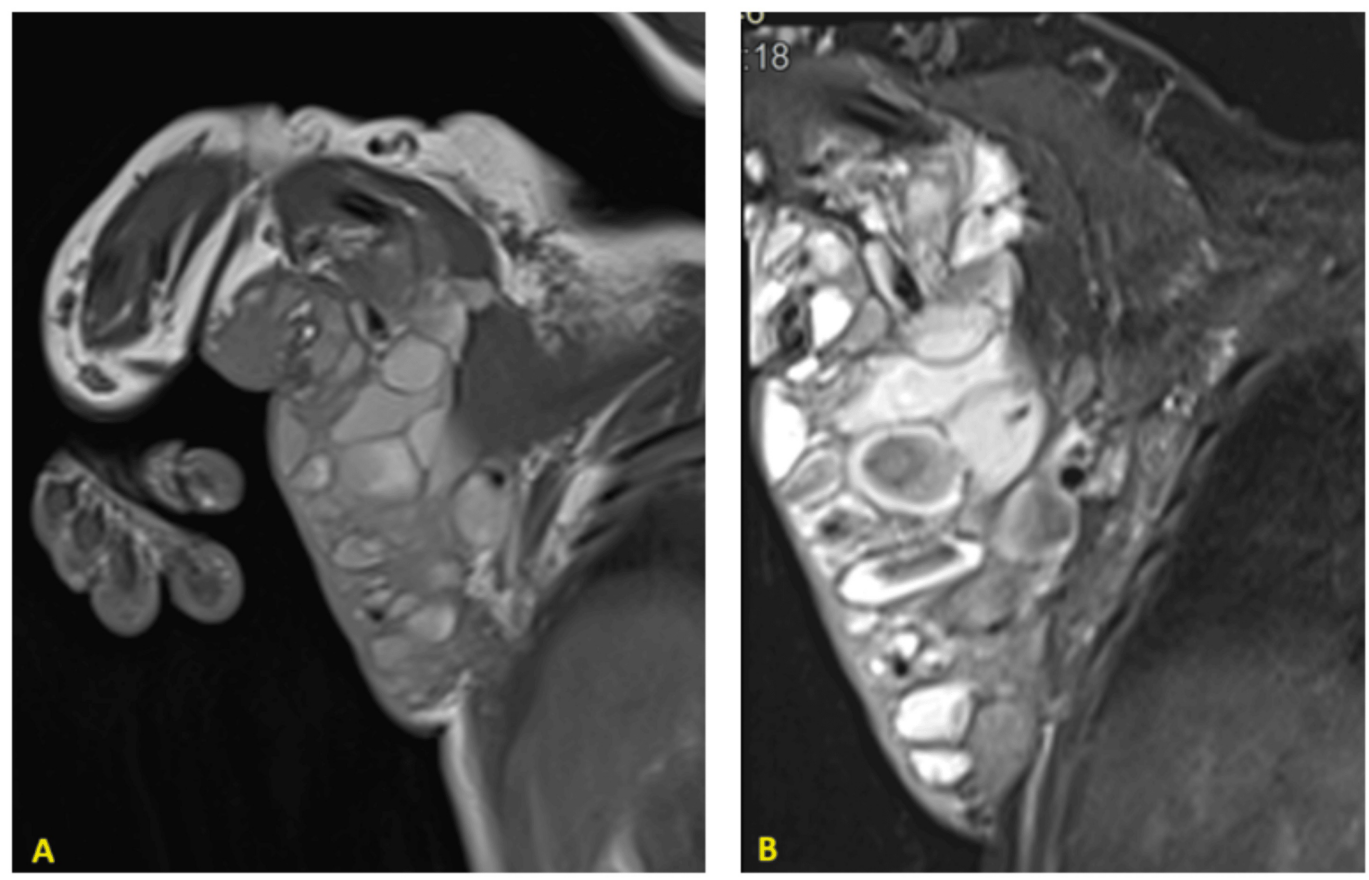
Coronal MRI Sequences of the Upper Limb and Thorax (A) T1 coronal sequence without fat saturation: multilocular lesion containing an intermediate hyperintensity, adjacent to the right lateral thoracic wall and the homolateral axillary recess (B) T2 coronal sequence with fat saturation: the loculations exhibit marked hyperintensity on T2-weighted imaging, with some showing hypointensity on T2, indicative of slow flow

**Figure 3:**
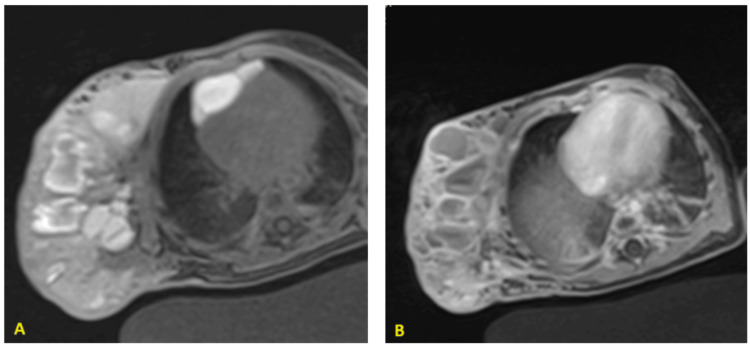
Axial MRI Sequences of the Upper Limb and Thorax A: Axial T1 sequence with fat saturation without contrast agent injection: Evidence of an anterior mediastinal lesion communicating with the previously described lesion and exhibiting similar signal characteristics B: Axial T1 sequence with fat saturation after contrast agent injection: Peripheral enhancement observed in some loculations, with progressive endoluminal enhancement noted in others

MRI demonstrated a bulky mass involving the right hemithoracic wall and extending into the ipsilateral upper limb, connected to an antero-superior mediastinal mass suggestive of hemangiomas based on its morphology and blood flow characteristics (Figures 2,3). A biopsy of the mass confirmed a histological diagnosis consistent with capillary hemangioma.

Treatment was initiated immediately after birth with prednisolone at 3 mg/kg/day, maintained for two weeks before being gradually tapered. This approach was combined with salicylic acid at 10 mg/kg/day. Due to the unavailability of sirolimus initially, everolimus was administered at 0.1 mg/kg/day for three months, after which the therapy was transitioned to sirolimus, which has been continued to date. Additionally, adjuvant therapy with calcium and potassium was provided, and the patient has been closely monitored by the pediatric dermatology team. However, the patient experienced one episode of peri-anal herpetic infection, effectively managed with acyclovir.

**Figure 4:**
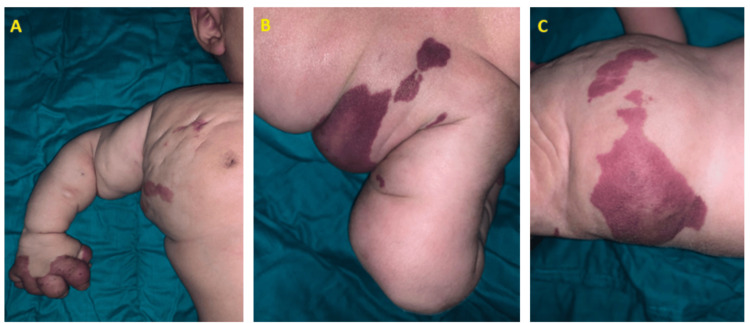
Volume Evolution of the Mass After Six Months

After initiating treatment, the patient demonstrated a favorable clinical response. Within three months, the mass reduced by over 50% of its original size and the lesion became noticeably paler (Figure 4).

Additionally, thrombocytopenia and anemia resolved, and the coagulation abnormalities improved significantly.

**Figure 5:**
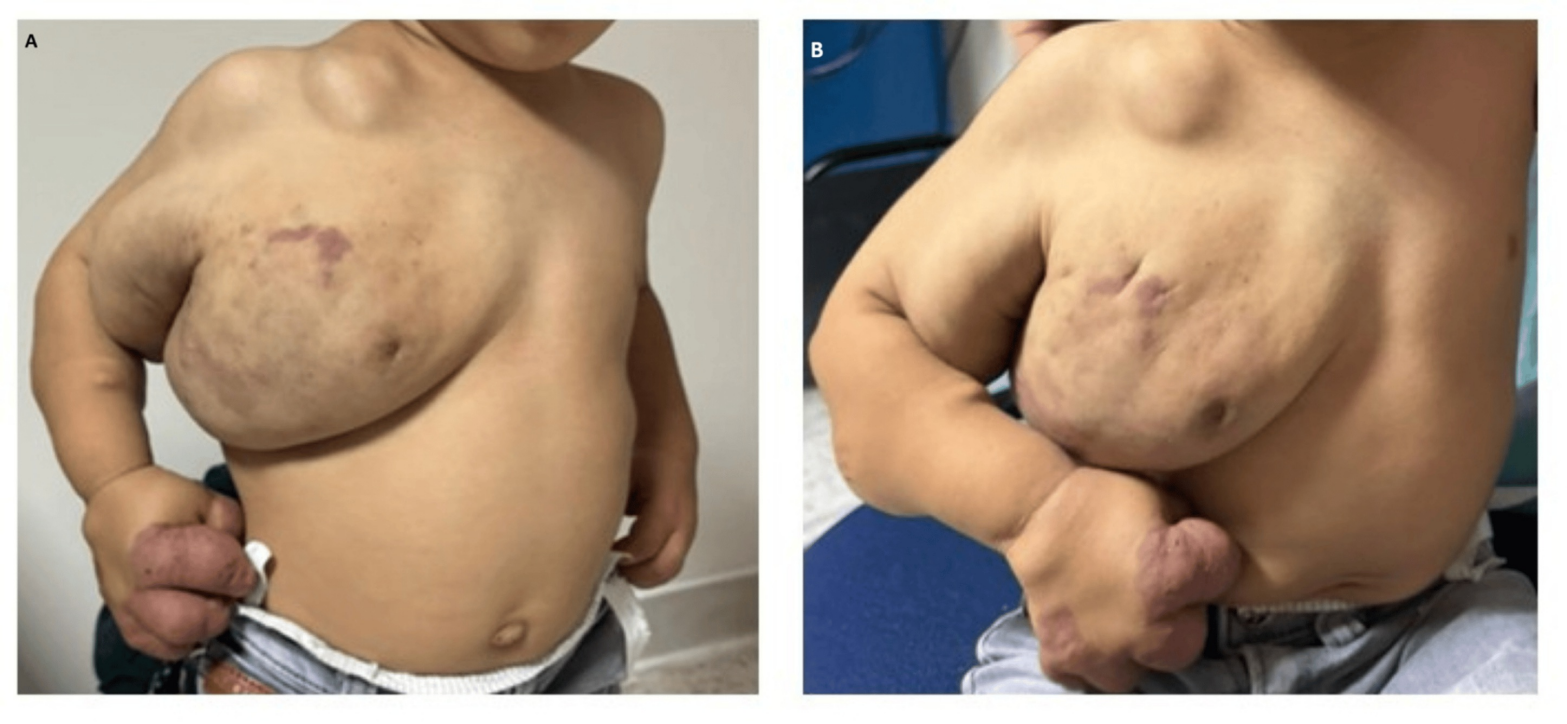
Evolution of the Mass at 2-Year Follow-Up

The patient is currently two years old and continues to be monitored with clinical and biological evaluations every six months. The volume of the mass has stabilized, and the lesion has become completely pale, indicating a positive therapeutic response. Additionally, the patient exhibits normal growth in both stature and weight, along with appropriate psychomotor development (Figures 5).

## Discussion

Kasabach-Merritt Syndrome (KMS), first identified by Kasabach and Merritt in 1940, is a rare hematologic disorder characterized by a large hemangioma accompanied by severe thrombocytopenia and hypofibrinogenemia [5]. Patients often present with anemia and elevated D-dimer levels. Notably, KMS is exclusively linked to two vascular tumors: kaposiform hemangioendothelioma (KHE) and tufted angioma (TA), both of which belong to the same neoplastic spectrum [1].

KHE typically manifests as a progressively enlarging, firm, solitary purpuric lesion, affecting either cutaneous or soft tissue [6]. It predominantly involves the extremities, though it can also appear on the trunk and cervicofacial region. The retroperitoneum is the most frequently affected extracutaneous site, followed by muscles, bones, and the thoracic cavity. Notably, approximately 10% of individuals with KHE do not exhibit skin involvement.

Tufted angioma, by contrast, is primarily found on the trunk and presents as violaceous macules and plaques [7].

KMS is commonly associated with a rapidly growing and potentially painful tumor. It is distinguished by severe thrombocytopenia and consumptive coagulopathy, including hypofibrinogenemia. Given these hematologic abnormalities, laboratory evaluation is essential for diagnosis and includes a complete blood count, fibrinogen levels, D-dimer levels, prothrombin time (PT), and activated partial thromboplastin time (aPTT).

Patients with KMS exhibit profoundly low platelet counts, typically ranging from 3,000 to 60,000 per microliter [8]. Fibrinogen levels are significantly reduced, while D-dimer and fibrin degradation products are markedly elevated. PT and aPTT values are generally normal to slightly prolonged. Additionally, substantial anemia may arise due to intralesional bleeding, coagulopathy, sequestration of blood within the tumor, or hemolyticanemia caused by shear stress on red blood cells within the aberrant tumor vasculature. When platelet levels drop below 10,000 per microliter, widespread petechiae are frequently observed [8].

In our case, we observed a mass with a heterogeneous erythematous-violaceous, orange peel-like appearance. It was warm and firm upon non-pulsatile palpation. While the mobility of the shoulder, elbow, wrist, and fingers remained intact, it was restricted by the presence of the mass, which was located in the right hemothorax and right upper limb. The condition developed in an apyretic context and was accompanied by respiratory distress. Apart from these findings, the remainder of the clinical examination was unremarkable.

MRI is the primary imaging modality used to assess vascular tumors associated with KMS. These tumors typically present as diffusely enhancing masses, appearing isointense to muscle on T1-weighted images and hyperintense on T2-weighted images [9]. They frequently extend across multiple tissue planes and exhibit poorly defined margins.

Characteristic MRI findings include cutaneous thickening and fat stranding. Superficial draining vessels often appear dilated, while intratumoral vessels tend to be small and sparse. Additionally, signal voids suggestive of hemosiderin deposits are commonly observed [9].

In our case, the diagnosis was determined through a comprehensive assessment, incorporating the characteristic elementary lesions, identified biological abnormalities, MRI findings, and biopsy results.

The treatment of KMS focuses on two key objectives: managing coagulopathy and thrombocytopenia, and eliminating the hemangioma [10]. Although thrombocytopenia is often severe at presentation, platelet transfusions should be reserved for cases of active bleeding or for surgical and procedural preparation. Due to their short circulatory lifespan, infused platelets have been observed to potentially accelerate tumor growth and, in some instances, exacerbate KMS [11].

Cryoprecipitate may be administered in cases of active bleeding, for surgical preparation, or when fibrinogen levels fall below 100 mg/dL. However, it is not recommended for asymptomatic patients or those with only mildly reduced fibrinogen levels. Packed red blood cells (PRBC) should be transfused solely for symptomatic anemia. Heparin is contraindicated in KMS.

Additionally, effective pain management is crucial, as tumor engorgement with blood elements can result in severe pain and significant mass effects on surrounding tissues [8].

Complete surgical resection offers the most definitive treatment for small, localized tumors, as well as those that have diminished in size following medical therapy or pose a life-threatening risk. However, at the initial presentation of KHE, surgical intervention is rarely viable due to the tumor’s infiltrative nature and the presence of coagulopathy [12].

Additionally, radiation therapy has been reported as a treatment option for KMP [13,14], and successful embolization has been documented in patients with KMP [15].

Medical management of KMS has involved various agents, including corticosteroids, alpha interferon (IFN), vincristine (VCR), and other chemotherapy drugs, administered either individually or in combination [16,17], with varying degrees of effectiveness.

Corticosteroids, given at doses ranging from 2-30 mg/kg/day, have been shown to improve hematological parameters in 10% to 30% of patients within days of initiation, though they do not significantly impact tumor size [8]. IFN, whether used alone or alongside corticosteroids, has demonstrated success in resolving coagulopathy and promoting tumor regression in approximately 40% of cases [8].

In a retrospective study, 15 patients received VCR as first-line therapy, either alone or in combination with other agents. All patients demonstrated improvements in coagulation and hematologic parameters. Furthermore, 13 of them showed significant tumor size reduction following VCR treatment [18].

Sirolimus, with or without steroids, is a viable therapeutic option that eliminates the need for central line placement, as both medications are administered orally. Regular blood monitoring is necessary to assess sirolimus levels and adjust the dosage accordingly to maintain the target concentration. While these regimens may introduce steroid-related side effects, they also carry risks associated with sirolimus, including immunosuppression, mucositis, and dyslipidemia [19].

In our case, the initial combination of everolimus, corticosteroids, and salicylic acid led to a favorable evolution, characterized by significant regression of the mass by more than 50%, resolution of thrombocytopenia, and correction of coagulation disorders. However, after three months, once available, sirolimus was introduced to replace everolimus, further contributing to the continuation of therapeutic benefits.

## Conclusions

KMS is a rare yet serious disorder that demands early detection and a multidisciplinary approach to ensure optimal patient outcomes. Due to its unpredictable progression, treatment must be tailored to both the underlying vascular tumor and the consumptive coagulopathy responsible for platelet depletion and clotting abnormalities.

Advancements in therapeutic interventions have brought promising improvements in management. While surgical resection remains the most definitive option for select cases, medical therapies have proven invaluable in stabilizing patients and mitigating disease impact.

Looking ahead, ongoing research and clinical expertise will continue to refine treatment strategies and enhance survival prospects. By fostering collaboration among specialists in hematology, oncology, dermatology, radiology, and surgery, the medical community can advance diagnostic accuracy, expand therapeutic options, and ultimately improve outcomes for individuals affected by KMS.
